# Using Communication Accommodation Theory to Improve Communication Between Healthcare Providers and Persons With Dementia

**DOI:** 10.7759/cureus.30618

**Published:** 2022-10-23

**Authors:** Beheshta Momand, Olivia Sacuevo, Masuoda Hamidi, Winnie Sun, Adam Dubrowski

**Affiliations:** 1 Health Sciences, Ontario Tech University, Oshawa, CAN

**Keywords:** simulation-based training, quality of care provided, healthcare providers' knowledge, communication, dementia

## Abstract

The ability of healthcare workers to communicate effectively with dementia patients is critical in the healthcare context. This is because persons with dementia have difficulty expressing their views due to cognitive and language impairments. Therefore, it becomes essential that healthcare workers obtain the necessary training to handle the needs and concerns of persons with dementia. Furthermore, when the severity of the illness worsens, people with dementia may find it difficult to communicate verbally, so they rely heavily on nonverbal communication. Nonverbal communication is very useful for indicating pain and suffering. Identifying these nonverbal indicators by health experts allows them to begin treatment sooner, ultimately increasing the quality of life. Studies have found simulations to be an effective way of educating health professionals in the development/improvement of communication skills; however, they lack the capacity to identify and act on specific nonverbal signs. This editorial suggests that using communication accommodation theory (CAT) could be an effective tool for teaching communication skills to health professionals. CAT can give a framework for an improved understanding of nonverbal indications in dementia patients and strategies for healthcare practitioners to alter and use that information in patient care.

## Editorial

Introduction

Dementia is a general term for the loss of memory, language, problem-solving, and other thinking abilities that are severe enough to interfere with daily life [1]. According to Perry et al. (2005), the prevalence rates for dementia in adults aged 65 remain at 5%-8% in North America, while those 85 years of age are 35%. In other words, the chance of developing dementia rises as people age [1]. Recent studies claim that healthcare professionals and dementia patients cannot effectively communicate verbally or nonverbally due to a lack of knowledge [1]. As a result, it becomes challenging for healthcare professionals to know when to alter their speech, address patients' needs and concerns, and deliver critical information effectively [1]. Health professionals must communicate effectively with patients, especially those diagnosed with dementia. This is because dementia affects interactions with healthcare providers and family members due to their cognitive, behavioral, and linguistic challenges [1].

Effective communication between healthcare providers and patients is crucial for achieving high-quality treatment and patient satisfaction [1]. The process of exchanging ideas, concerns, and information with others at a particular time and location is called effective communication. Communication can be both verbal and nonverbal. Verbal communication includes written or spoken language, such as words or phrases communicated. Nonverbal or linguistic communication is referred to as messages or signals delivered via facial expressions, gestures, posture, and body language. Without effective communication, the quality of treatment can suffer, as can resource misallocation, increased healthcare costs, and death in certain situations [1].

Communication challenges in dementia patients

Communicating with dementia patients poses a variety of difficulties. Some of these difficulties are connected with varying degrees of impairment, such as memory loss, reduced attention span, and impaired judgment, insight, and reasoning [1]. Individuals may also begin to lose touch with the present and misinterpret what is happening around them. The combination of deficiencies causes communication problems, making it difficult for health practitioners to speak with and care for patients. For example, people with dementia may display repetitive speech, misunderstand language, or make half-comments [1]. In these cases, healthcare staff may be confused about what the individual is saying or how to approach them. Health workers may break down lengthy statements, use simple vocabulary, and give people time to respond, all of which are helpful ways to communicate verbally with people who have dementia.

In addition to verbal communication, people with dementia also exhibit several nonverbal communication. This is partly because as the severity of the condition worsens, so does the person's capacity to communicate verbally. For example, individuals who cannot communicate verbally may communicate using nonverbal signs such as facial expressions, body gestures, verbalization, and mental state changes [2]. Nonverbal signs are also employed in many circumstances where people are in pain and unable to communicate verbally with their caregiver. Facial grimacing, distress gestures, groaning, sobbing, wrath, or refusing meals are some nonverbal indications of pain in dementia patients [2]. As a result, it is up to health professionals who care for people with dementia to make the required efforts to interpret nonverbal cues.

Poor communication between a healthcare provider and a dementia patient can result in disagreements, isolation, and depression. Furthermore, failing to recognize nonverbal pain cues significantly impacts their quality of life and can lead to poor medical results [1]. Therefore, it is essential for healthcare personnel to have the required verbal and nonverbal abilities to communicate successfully with people who have dementia.

The current gap in health provider and persons with dementia communication training

Simulation is one successful method for improving communication between healthcare personnel and dementia patients [3]. Simulation is a sort of experiential learning that allows individuals to practice skills and knowledge in a simulated environment. According to research, simulation-based learning can efficiently grow health professionals' knowledge, abilities, and attitudes while protecting them from unnecessary dangers; however, present frameworks targeted toward persons with dementia focus on verbal communication rather than nonverbal communication [3]. For example, the VERA framework, which stands for validation, emotion, reassurance, and activity, was developed to encourage compassionate reactions between healthcare workers and dementia patients [4]. According to the VERA framework, fine-tuning behaviors and "connecting with the patient" may establish a caring atmosphere that fosters increased communication. The problem with this framework is that it does not address the nonverbal cues required to care for dementia patients. For example, health practitioners may struggle to decipher the meanings of suspected facial expressions, body gestures, certain tones of voice, and interpersonal interactions [4]. A framework that allows healthcare professionals to recognize and distinguish the elements of nonverbal cues that relate to pain will help accurately fill the needs of dementia patients.

Proposed solution

To date, there are no theory-based models for simulation-based training for healthcare providers focused on verbal and nonverbal communication when conversing with dementia patients. One theoretical framework that can be used to improve communication for this population is the communication accommodation theory (CAT). CAT is concerned with behavioral changes individuals make to attune their communication with the individual they are conversing with and the degree to which people perceive their partners are attuning to them [5]. The basis of the theory lies in the idea that individuals attune their communication to accommodate their style of speech to the individual they are conversing with. Some advantages of CAT are increased communication productivity, improved positive social identity, and the sender gaining approval from the receiver [5].

CAT emphasizes two critical ideas, called convergence and divergence. Convergence refers to the strategies individuals take to adapt their communicative behaviors (verbal or nonverbal) to the individual they are conversing with to reduce social differences and create a comfort zone [5]. On the other hand, divergence accentuates the idea of verbal and nonverbal differences in communication [5]. Thus, generally speaking, CAT relates to the social identity, language, and environment of the persons participating in the communication [5]. Furthermore, CAT concerns how the communicator adapts to a broader social environment and how the communicator and the message recipient interact during the communication process.

To further demonstrate CAT, consider the case of a 65-year-old patient with dementia. This individual may exhibit clenched teeth, sighing, tight body posture, or seemingly annoyed. All of these nonverbal indications point to the patient being in pain. A health practitioner trained in CAT may be better equipped to identify verbal/nonverbal cues. By recognizing and addressing these problems, healthcare providers may respond more quickly, thus improving the patient's health. The concept of convergence in CAT demands health providers to speak clearly and deliberately, repeat statements, maintain eye contact, be patient and empathetic, avoid judgment, and give comfort. During divergence, the health practitioner would communicate his or her dissatisfaction with the patient by speaking too rapidly, overloading them with information, and failing to listen to them. Therefore, we suggest that CAT has the potential to be included in the simulation-based training program to address the communication challenges of persons with dementia.

Conclusion

Communication is a fundamental component in the delivery of care. It allows individuals to express their concerns and make connections with their environment. Without effective communication, the quality of care delivered to patients may be impacted, resulting in increased healthcare costs, resource misallocation, and, in some cases, mortality [1]. This is especially important in patients who have dementia because dementia is associated with a decline in cognitive and linguistic functions, making communication difficult [1]. In addition, persons suffering from pain due to severe dementia communicate primarily through nonverbal communication. As a result, health practitioners must communicate effectively and address any issues persons with dementia may have. Current studies have suggested that simulation-based training effectively teaches verbal and nonverbal communication [3]. However, existing frameworks for improving communication between healthcare providers and people with dementia are mostly based on verbal rather than nonverbal communication. For example, the VERA framework was one method that helped healthcare workers establish familiar ground and use compassion as a form to communicate with dementia patients [4]. Unfortunately, this method was ineffective for long-term purposes since attuning with dementia patients can be challenging when their mood alters into irritation or aggression, making the technique less practical to utilize in those circumstances. We argue that employing CAT can improve the communication challenges that healthcare providers face when conversing with dementia patients, untimely improving the quality of care delivered.
